# Alga-based mathematical model of a life support system closed in oxygen and carbon dioxide

**DOI:** 10.18699/VJGB-23-101

**Published:** 2023-12

**Authors:** D.A. Semyonov, A.G. Degermendzhi

**Affiliations:** Institute of Biophysics of the Siberian Branch of the Russian Academy of Sciences, Federal Research Center “Krasnoyarsk Science Center SB RAS”, Krasnoyarsk, Russia; Institute of Biophysics of the Siberian Branch of the Russian Academy of Sciences, Federal Research Center “Krasnoyarsk Science Center SB RAS”, Krasnoyarsk, Russia

**Keywords:** life support system (LSS), mathematical model, mixed culture of two algae, система жизнеобеспечения (СЖО), математическая модель, смешанная культура двух водорослей

## Abstract

The purpose of the study was to compare quantitative analysis methods used in the early stages of closed-loop system prototyping with modern data analysis approaches. As an example, a mathematical model of the stable coexistence of two microalgae in a mixed flow culture, proposed by Bolsunovsky and Degermendzhi in 1982, is considered. The model is built on the basis of a detailed theoretical description of the interaction between species and substrate (in this case, illumination). The ability to control the species ratio allows you to adjust the assimilation quotient (AQ), that is, the ratio of carbon dioxide absorbed to oxygen released. The problem of controlling the assimilation coefficient of a life support system is still relevant; in modern works, microalgae are considered as promising oxygen generators. At the same time, modern works place emphasis on empirical modeling methods, in particular, on the analysis of big data, and the work does not go beyond the task of managing a monoculture of microalgae. In our work, we pay attention to three results that, in our opinion, successfully complement modern methods. Firstly, the model allows the use of results from experiments with monocultures. Secondly, the model predicts the transformation of data into a form convenient for further analysis, including for calculating AQ. Thirdly, the model allows us to guarantee the stability of the resulting approximation and further refine the solution by small corrections using empirical methods.

## Abstract (ru)

Целью исследования было сравнить методы количественного анализа, применявшиеся на ранних этапах создания прототипов замкнутых систем, с современными подходами анализа данных. В качестве примера рассмотрена математическая модель устойчивого сосуществования двух микроводорослей в смешанной проточной культуре, предложенная Болсуновским и Дегерменджи в 1982 г. Модель построена на основе детального теоретического описания взаимодействия видов и субстрата (в данном случае освещенности). Возможность управления соотношением видов позволяет регулировать ассимиляционный коэффициент (AQ), т. е. отношение поглощенного углекислого газа к выделенному кислороду. Задача управления ассимиляционным коэффициентом системы жизнеобеспечения до сих пор актуальна, микроводоросли рассматриваются как перспективные генераторы кислорода и в современных работах. При этом акцент в них сделан на эмпирических методах моделирования, в частности на анализе больших данных; также работы не выходят за пределы задачи управления монокультурой микроводорослей. В настоящем исследовании мы обращаем внимание на три результата, по нашему мнению, удачно дополняющих современные методы. Во-первых, модель позволяет использовать результаты экспериментов с монокультурами, во-вторых, предсказывает преобразование данных к виду, удобному для дальнейшего анализа, в том числе для вычисления AQ. В-третьих, модель позволяет гарантировать устойчивость полученного приближения и в дальнейшем искать решение как малую поправку эмпирическими методами.

## Introduction

Nowadays, complex systems are predominantly viewed as a “black box” generating large amounts of data. The development of relevant methods for big data analysis has been facilitated by a significant increase in the availability of data recording methods and a decrease in the cost of computing power. When designing closed life support systems, data continue to be scarce and expensive. Theoretical approaches based on detailed descriptions of the components of complex systems can predict useful approaches to data preprocessing. Mathematical models, seeking to describe a complex system in aminimally complex way, transform an array of experimental data into a form convenient not only for analysis, but also for perception by a human operator. In addition, mathematical models help solve problems that are still relevant today. We illustrate these points using the example of controlling the assimilation quotient (AQ) of a mixed culture of two algae.

Can we learn anything from the early experience of prototyping closed circuit life support systems (CLSS)? The history of creating closed life support systems goes back more than half a century. Due to the revival of interest in creating bases on the Moon and Mars in the last decade, the relevance of this area of work has increased markedly (Keller et al., 2021; Liu et al., 2021). Since at the initial stages some prototypes were created and studied in detail, later rejected for various reasons, there is a desire to study the experience of these works for possible use in modern projects. Most modern publications persistently propose universal approaches to creating individual life support modules and testing the system (Heinicke, Verseux, 2023; Metelli et al., 2023). Can old approaches be useful for new projects? Also, there is a temptation to compare the approaches used then with those common now, in particular with big data analysis methods.

It is convenient to conduct a similar mental experiment at a preliminary stage for a system that has a fairly detailed theoretical description. In our case, this is a system for co-cultivating two algae (Chlorella vulgaris and Spirulina platensis) used as an oxygen generator for life support systems (LSS). The idea of using algae to create life support systems is still relevant (Häder, 2020; Fahrion et al., 2021; Matula et al., 2021; Keller et al., 2023). In particular, Chlorella vulgaris and Spirulina platensis are still actively considered as promising species for this task (Helisch et al., 2020; Cycil et al., 2021; Matula, Nabity, 2021; Matula et al., 2021). We cannot confidently say that all the authors of these works are sincerely convinced of the future role of microalgae in LSS. We believe that higher plants are more promising for solving the problem of providing humans with oxygen and food. However, we, like perhaps many of the authors listed, consider microalgae to be a successful teaching aid. Due to a number of advantages, the cultivation of microalgae is a good model object. For example, in relatively recent literature one can find works devoted to the management of microalgae monocultures (Hu et al., 2008, 2012, 2014), which demonstrate the effectiveness of various management methods. That is, the theoretical work on managing microalgae cultivation in a series of three articles is methodological in nature. We see an opportunity to complement this series of articles by turning to the analysis of the model of forty years ago. As part of the work to create closed life support systems, in 1982, a model for managing a mixed flow-through culture of two algae was created (Bolsunovskiy, Degermendzhi, 1982).

The use of algae as the only autotrophs in the life support system allows us to apply a convenient simplification to reasoning about the stoichiometry of oxygen reduction and carbon dioxide sequestration in an algal cultivator. To a first approximation, we can assume that all carbon dioxide is released by the human body in the oxidation reactions of carbohydrates and fats. This assumption is based on the fact that the use of amino acids by the human body as a significant source of energy is possible with an unbalanced diet, excessive physical activity, or with certain chronic diseases. Having ruled out these three possibilities, we will assume that amino acids make a negligible contribution to respiration. Carbohydrates and fats are the main sources of energy for the human body and the main products of algae biosynthesis.

Another convenient simplification would be to ignore the synthesis of amino acids by algae. Unfortunately, the biomass composition of both algae indicates that proteins are present in large quantities. However, we can allow a first approximation, which should be followed by adjustments to the model if it is necessary to close the nitrogen exchange. That is, to a first approximation, as much as a person oxidizes carbohydrates and fats, the same amount of carbohydrates and fats should be synthesized by algae to bind excess carbon dioxide and regenerate the oxygen used by the person. The use of higher plants would not allow us to resort to such a simple first approximation, since in addition to carbohydrates, fats and proteins, the composition of higher plants contains lignin in noticeable quantities, which differs significantly in stoichiometry from both carbohydrates and fats.

Depending on the diet and level of physical activity, the human body can use different substrates to obtain energy. With sufficient oxygen availability, the main source of energy is the oxidation of fatty acids in mitochondria. When there is a lack of oxygen, the human body prefers carbohydrates as the main source of energy. Thus, the ratio of carbon dioxide emitted by a person and oxygen absorbed can vary from almost 0.7 (oxidation of fats) to 1.0 (oxidation of carbohydrates). For a person, there is even a possibility of a short-term excess of the respiratory index of 1.0 as a result of intense physical activity (acidosis with loss of bicarbonates) and even a long-term excess under the condition of carbohydrate nutrition and an increase in body weight with the accumulation of fat. Unlike humans, algae, on average, maintain a relatively constant composition during their life cycle. Since no synchronization or fluctuations in abundance were observed in the analyzed flow culture, it is possible to use average values of assimilation indices for each of the two algae species.

Assimilation indices reflect the stoichiometric proportion in which the bound carbon dioxide molecules relate to the produced oxygen molecules. Since we agreed to describe the entire metabolism as a first approximation by the balance of fats and carbohydrates, we will leave outside the scope of this article the study of the possibility of shifting the assimilation index of algae by variations in nitrogen nutrition (Belyanin et al., 1980, p. 114–117). We will consider the situation with nitrogen nutrition to be stable and assume that the assimilation index of a system of two algae can vary within the limits indicated in the literature. The metabolic constancy of autotrophs and human metabolic plasticity must somehow be reconciled within the framework of the work of the CLSS. The range of possible total assimilation index of two algae limits the diet and metabolic activity of a person settled in the CLSS. An important assumption will be that we can adhere to the average specified range by rationally managing a person’s diet and physical activity. Then, for example, depending on a long-term increase in the level of physical activity, a person’s respiratory coefficient may shift, which will require a shift in the assimilation quotient of the life support system. The design of the life support system should allow for the ability to adapt to the needs of human metabolism. In the analyzed model, we will be interested in the possibility of controlling the composition of a mixed algae culture and controlling the total assimilation quotient.

## Materials and methods

Assessment of assimilation indices of a mixed culture of two algae. In order to imagine in more detail the processes of gas exchange in the system under study, we will use the gross formulas of the biomass of chlorella (C6.0H9.7O2.635N0.937) (Belyanin et al., 1980, p. 111) and spirulina (C6.0H10.84O2.06N0.87) (Belyanin et al., 1980, p. 116). Since the system is considered not closed in nitrogen at the first stage, it is possible to simplify the formulas by considering that the main form of nitrogen absorption by algae is urea or ammonium ions, and also by removing oxygen in the form of water from the formulas. We obtain the residue in the form (С6.0H1.6) for chlorella and (C6.0H4.11) for spirulina. So, it turns out that the synthesis of chlorella and spirulina biomass allows one absorbed liter of carbon dioxide to release 1.13 liters and 1.3425 liters of oxygen, respectively, which corresponds to the assimilation quotients AQ = 0.885 for chlorella and AQ = 0.745 for spirulina.

The assimilation index of a mixed culture can be easily obtained from the mass ratios of algae in the culture:

**Formula. 1.**
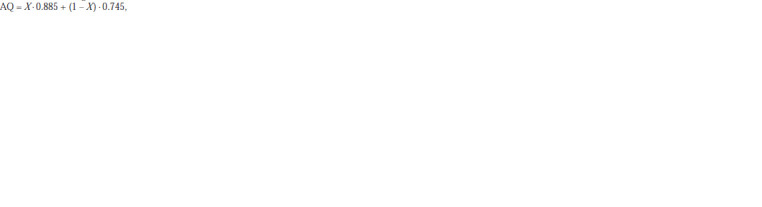
Formula 1

where X is the proportion of spirulina in the culture. So, for the initially obtained stable mixed culture X = 0.6 and AQ = 0.6 · 0.885 + 0.4 · 0.745 = 0.829. Controlling the composition of a mixed culture makes it possible to obtain an AQ value ranging from 0.745 (Spirulina monoculture) to 0.885 (Chlorella monoculture).

Mathematical model. In order to predict the stationary state of algae populations in a flow cultivator, a mathematical model that summarizes information about the influence of control factors on a system of two species is needed. It is precisely this model of a flow cultivator with two algae that was built in (Bolsunovskiy, Degermendzhi, 1982). The model describes the coexistence of two species competing for a limiting substrate. The limiting substrate in this case is the luminous flux. In the model, there is a region of illumination parameters in which two species stably coexist; in addition, there are areas of dominance for each species, when the competing species is forced out. Of course, there is also a range of parameters that does not allow any of the species to reproduce; they are simply washed out of the cultivator with a given flow and insufficient lighting. The flow of the substance in the cultivator was stabilized by recording the absorption of chlorophyll at a wavelength of 680 nm, that is, the system maintained a constant optical density of the medium. The system can be controlled by adjusting the flow rate (that is, the optical density of the medium in the cultivator) and the light intensity. The model does not take into account the photoinhibition of spirulina growth at high light intensity, as well as the effects of metabolic inhibition at high population densities. The mathematical part of the model was obtained as a result of a quantitative description of experiments (Belyanin, Bolsunovskiy, 1980) using differential equations with the subsequent linearization procedure (Bolsunovskiy, Degermendzhi, 1982).

The model is a system of two differential equations, each of which reflects the population dynamics of one alga. The equations look like:

**Formula. 2.**
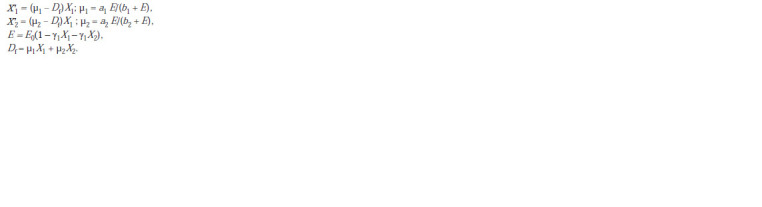
Formula 2

**Fig. 1.**
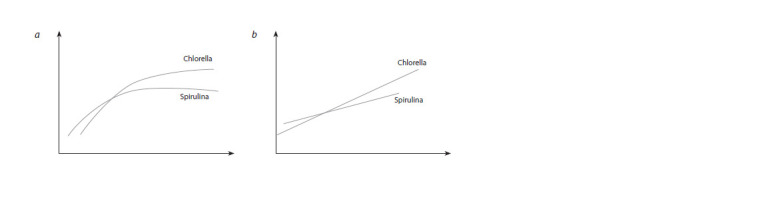
Specific growth rate depending on illumination of monocultures of chlorella and spirulina (a). Representing the same data in inverse coordinates (b) shows good agreement with the Michaelis–Menten equation.

E is average illumination, taking into account the absorption of light by algae cultures. E was obtained after expansion into a Taylor series and discarding nonlinear terms, taking into account the low optical density of the mixed culture. Df is the flow rate, which in further analysis is replaced by the optical density of the culture as an experimentally measured value. The equations reflect competition for light as a substrate. This substrate, as is known from experimental data on monocultures, is absorbed according to the Michaelis– Menten equation. Specific growth curves in monocultures demonstrate that Spirulina is more efficient at light uptake at low light levels, while Chlorella is more efficient at high light levels (Fig. 1).

In the parameter ranges characteristic of a stable joint culture of two algae (low population density and low light flux), the model should give the smallest discrepancy with experimental data. To change the ratio of species in the cultivator under these conditions, a small change in the lighting regime or a corresponding change in the flow is sufficient. Long-term increases and decreases in oxygen demand in the CLSS can be compensated by appropriately scaling the cultivator.

## Results

The first impression is that the culture of two practically noninteracting species, when competing for a single common substrate, should lead to a stable state when one species dominates and the other species is displaced. It turns out that it is possible to understand why coexistence occurs by carefully analyzing the interaction of species with the substrate in a monoculture. Chlorella not only does better in high light, but it also creates some advantage for Spirulina in a mixed culture compared to a monoculture. In fact, in the presence of chlorella, spirulina can exist in areas of higher light. Chlorella “shadows” spirulina, creating more comfortable conditions for it. Amore detailed analysis of the biology of these species made it possible to identify adaptations to high and low light levels, as well as adaptation to different spectral ranges (Bolsunovskiy, Degermendzhi, 1982). But even without taking into account this adaptability to different parts of the spectrum and, in fact, using the material of experiments with monocultures, it is possible to obtain non-trivial dynamics in the mixed culture model. A mathematical model helps move from qualitative explanation to quantitative predictions.

**Fig. 2.**
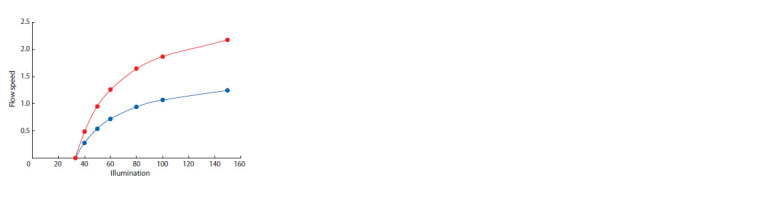
The area of existence of a stable culture of two algae is limited by two curves on the Illumination/Flow Speed plane.

The model allows us to obtain an area of sustainable coexistence of two species in a continuous culture. Graphically, the area is presented on a plane in the coordinates of illumination (E0) and the optical density of the crop at a wavelength of 680 nm (C), reflecting the flow rate in the cultivator (Fig. 2).

It is necessary to note that extrapolation of the model results to the area of high illumination and high density of culture is undesirable, since in this area the effect of factors not taken into account in the modeling has been experimentally shown (Belyanin et al., 1980, p. 32–48).

The model allows you to calculate stationary concentrations of components, that is, the population density of individual species:

**Formula. 3.**
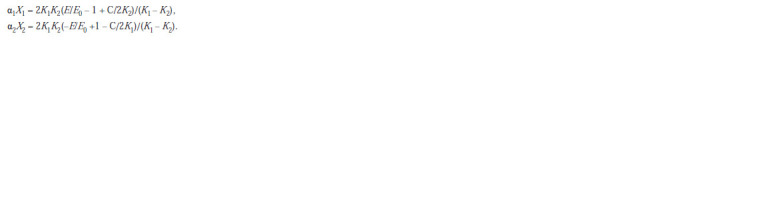
Formula 3

The steady-state concentrations of each algae species are designated in the model as X1 and X2. Since we assume that each alga is characterized by a strictly defined composition and AQ values, the total AQ is a simple superposition: AQ = (X1·AQ1+ X2·AQ2)/(X1+ X2). That is, the X1/X2 = const curves will simultaneously be curves with a constant value of the totalAQ. It seems paradoxical that all these curves intersect at one point, but the paradox is resolved simply because at this point X1 = X2 = 0. That is, it does not matter what the ratio of oxygen produced to carbon dioxide absorbed is if the rate of photosynthesis drops to zero.

For the task of controlling the gas composition in a gasprocessing facility, it is important to determine where the relation X1/X2 = const is satisfied. The mathematical model was intended to qualitatively explain the observed phenomenon, namely the stable coexistence of two species. One cannot expect an accurate prediction of equilibrium positions over the entire region of existence of the system, but the model can provide a good first approximation for solving such a problem in practice.

Such an approximate algorithm for searching for the equilibrium state of the system will serve as a “rough tuning knob.” Amore accurate selection of parameters can be carried out experimentally.

In order to understand how a model can be used to analyze experimental data, let’s imagine that there are data, but there are no theoretical ideas about how the system functions. Apragmatic approach would be to search for the transformation of curves that limit the region of existence into straight lines in new coordinates. Then all straight lines on this plane passing through the intersection point and lying in the area of existence of a mixed culture could be taken as X1/X2 = const. For example, for a given type of curve, an approximation could be a transformation of the form C(E) = K· ln(E) – const, where K and const would be selected using the least squares method.

**Fig. 3.**
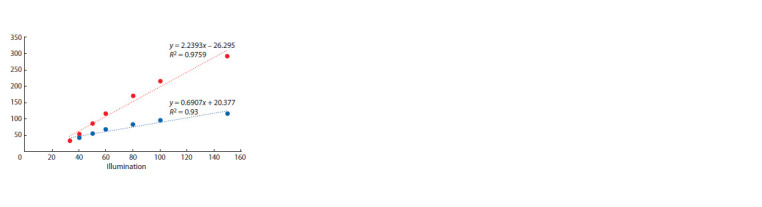
The result of the empirical selection of a transformation that “straightens” the data graphs in new coordinates.

Figure 3 shows the results of the inverse transformation of the E = exp(C/K + const) graphs. It can be noted that after the transformation the points are well approximated by a straight line.

All possible stable equilibrium positions of the system that allow the coexistence of two species can, after such a transformation, be represented by a bunch of straight lines passing through one point. For each such line we can take AQ = const. Since AQ is obtained by a simple superposition of the assimilation indices of two algae, it is natural to assume that on a plane where data on monocultures are represented by straight lines, data on a mixed culture will also be represented by straight lines.

It is worth paying attention to two obvious facts: (1) the chosen approximation is sensitive to the area in which the experimental data are collected; (2) the approximation produces a systematic error, underestimating the results at average illumination and overestimating them in the areas of low and high illumination.

**Fig. 4.**
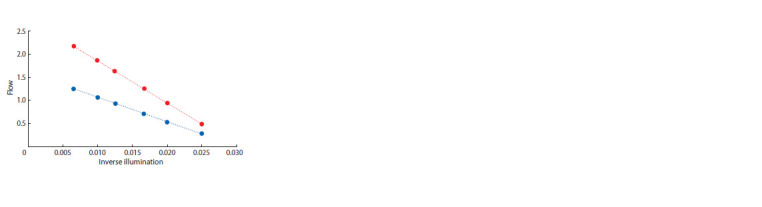
As follows from the model, to search for equilibrium positions in a mixed culture, it is convenient to present data in coordinates (Inverse Illumination/Flow).

Now let’s compare this approach with the one that follows from knowing the exact solution of the model. An exact approximation of the model solution will be given by transformation to coordinates (1/E0; C). Exact solutions are then converted to straight lines (Fig. 4). All points that obey the relations X1/X2 = const will also lie on straight lines passing through the common intersection point. It is this approximation that can be recommended for further use in processing experimental data as a first approximation.

Let’s imagine a situation where we have experimental data obtained under modern conditions. Let’s say a stationary state has been established in the cultivator. In the experiment, you can control the flow rate and illumination. Using gas analysis, you can obtain the AQ value for a stationary case, and then calculate the ratio of species in the culture. One can also imagine a direct measurement of the species ratio. Using modern methods, for example, flow cytometry, it is possible to automatically obtain data on the steady-state X1/X2 ratio. All this data can be used to restore the parameters of calibration graphs of the form X1/X2 = const. That is, the theory helps to choose a data preprocessing procedure for further analysis, for example, using methods of mathematical statistics, or artificial neural networks, or even in the form of graphical constructions. Moreover, the theory was obtained based primarily on data on the specific growth rate of algae in monocultures. Based on data on monocultures, empirical methods simply cannot predict the relationships in a mixed culture, so empirical methods, which include all modern “methods of big data analysis,” will require not only large, but also rather hard-to-access data.

How can we now determine the position of a straight line with a given ratio X1/X2? The bottom graph is the optical density of the spirulina monoculture, the top graph is the optical density of the chlorella monoculture. In order to find a position with a given ratio X1/X2 at a given level of illumination, it is necessary to divide the vertical segment connecting the lower and upper straight lines in the ratio X1/X2. The stability of the solution of the mathematical model guarantees that subsequent experimental refinement of the equilibrium position will be small. Empirical methods currently do not provide insight into the stability of the predictions obtained with their help.

## Conclusion

When creating complex biotechnological systems, fairly simple and visual mathematical models can be a good addition to modern methods of data analysis. If experimental data are difficult to access, the only way to predict the behavior of the system is to create an adequate mathematical model. In addition, in the case of closed life support systems, the ability to understand the structure of the system on the part of the human operator, as a rule, the occupant of this system, is important. The simpler and more obvious the mechanisms incorporated into the design of the life support system, the higher its reliability will be.

## Conflict of interest

The authors declare no conflict of interest.
